# Local Oxidative and Nitrosative Stress Increases in the Microcirculation during Leukocytes-Endothelial Cell Interactions

**DOI:** 10.1371/journal.pone.0038912

**Published:** 2012-06-14

**Authors:** Saptarshi Kar, Mahendra Kavdia

**Affiliations:** Department of Biomedical Engineering, Wayne State University, Detroit, Michigan, United States of America; Children’s Hospital Boston, United States of America

## Abstract

Leukocyte-endothelial cell interactions and leukocyte activation are important factors for vascular diseases including nephropathy, retinopathy and angiopathy. In addition, endothelial cell dysfunction is reported in vascular disease condition. Endothelial dysfunction is characterized by increased superoxide (O_2_^•−^) production from endothelium and reduction in NO bioavailability. Experimental studies have suggested a possible role for leukocyte-endothelial cell interaction in the vessel NO and peroxynitrite levels and their role in vascular disorders in the arterial side of microcirculation. However, anti-adhesion therapies for preventing leukocyte-endothelial cell interaction related vascular disorders showed limited success. The endothelial dysfunction related changes in vessel NO and peroxynitrite levels, leukocyte-endothelial cell interaction and leukocyte activation are not completely understood in vascular disorders. The objective of this study was to investigate the role of endothelial dysfunction extent, leukocyte-endothelial interaction, leukocyte activation and superoxide dismutase therapy on the transport and interactions of NO, O_2_^•−^ and peroxynitrite in the microcirculation. We developed a biotransport model of NO, O_2_^•−^ and peroxynitrite in the arteriolar microcirculation and incorporated leukocytes-endothelial cell interactions. The concentration profiles of NO, O_2_^•−^ and peroxynitrite within blood vessel and leukocytes are presented at multiple levels of endothelial oxidative stress with leukocyte activation and increased superoxide dismutase accounted for in certain cases. The results showed that the maximum concentrations of NO decreased ∼0.6 fold, O_2_^•−^ increased ∼27 fold and peroxynitrite increased ∼30 fold in the endothelial and smooth muscle region in severe oxidative stress condition as compared to that of normal physiologic conditions. The results show that the onset of endothelial oxidative stress can cause an increase in O_2_^•−^ and peroxynitrite concentration in the lumen. The increased O_2_^•−^ and peroxynitrite can cause leukocytes priming through peroxynitrite and leukocytes activation through secondary stimuli of O_2_^•−^ in bloodstream without endothelial interaction. This finding supports that leukocyte rolling/adhesion and activation are independent events.

## Introduction

Leukocyte-endothelial cell interactions and leukocyte activation are important factors for onset and progression of vascular diseases including nephropathy, retinopathy, cardiomyopathy, neuropathy and angiopathy [1], [2], [3], [4]. It is reported that the presence of leukocytes along the endothelium and the activation of leukocytes results in complications such as tissue edema and multiple organ failure [5], [6], [7]. Studies have shown that leukocyte-endothelial cell interactions are necessary for onset of microvascular dysfunction and tissue injury [8], [9], [10]. However, anti-adhesion therapies used to prevent vascular complications arising from leukocyte-endothelial cell interactions have not been very successful [7].

**Figure 1:**
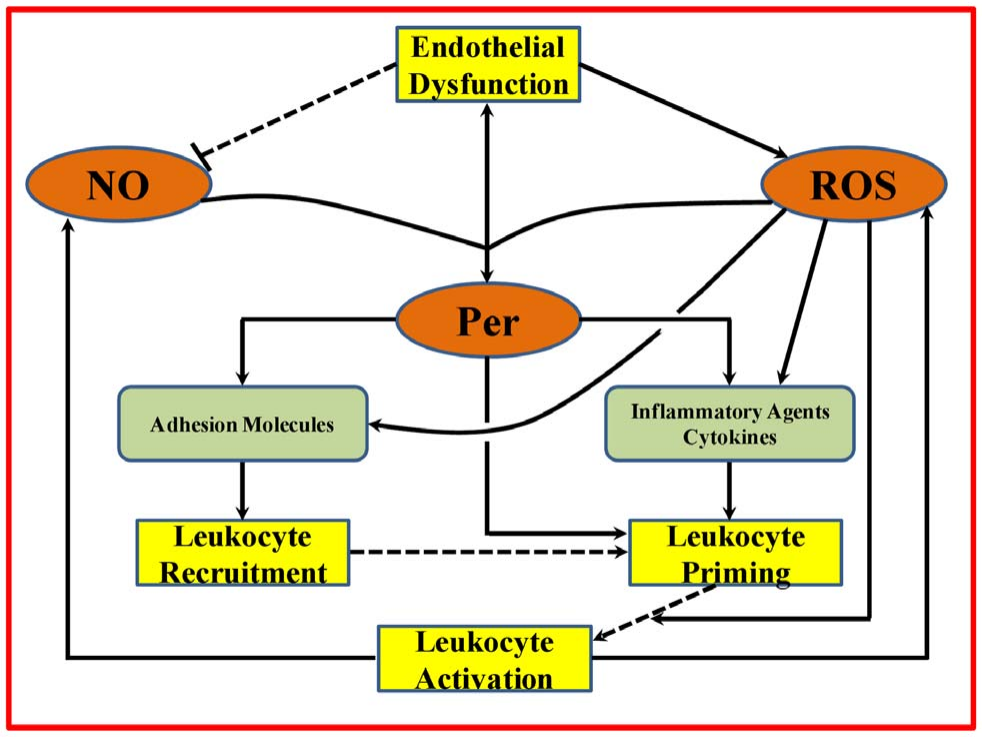
Schematic representation of the role of endothelial dysfunction on leukocyte related events through interactions between free radical species (NO, ROS and peroxynitrite). The free radical species are represented by the orange ovals, the leukocyte related events and endothelial dysfunction are represented by the yellow compartments and the chemical species expressed as a result of the interactions of free radicals (cytokines, adhesion molecules and inflammatory agents) are represented by the light green compartments. Endothelial dysfunction leads to increased ROS production from endothelium and a possible reduction in NO availability (indicated by the dashed lines). The ROS and NO combine to form peroynitrite (Per). ROS and peroxynitrite increase expression of adhesion molecules and cytokines leading to leukocyte recruitment and priming. Peroxynitrite and ROS can also prime and activate primed leukocytes, respectively. The dashed lines connecting the leukocyte related events shows the uncertaintly associated with their sequential nature.

Vascular disease conditions increase oxidative stress in endothelial cells, resulting in endothelial dysfunction [11]. As shown in Figure 1, endothelial dysfunction is characterized by increased superoxide (O_2_^•−^) production from endothelium and a reduction in NO bioavailability [11], [12]. There are many hypotheses for the reduction in NO bioavailability. The most important characteristic is a rapid reaction between NO and O_2_^•−^ to form peroxynitrite, which may reduce the NO availability even when NO production is increased under oxidative stress conditions [13], [14]. The increased oxidative stress increases cytokines, inflammatory agents and adhesion molecules expression on the endothelial cell surface and their ligands expression on the surface of the leukocytes [15], [16] resulting in recruitment of leukocytes to the endothelium [2], [15], [16], [17].

The process of leukocyte activation and transmigration is complex. Before activation of leukocyte, the leukocytes are converted to semi-activated state by a process of priming with the aid of priming agents [7], [18]. Priming agents include peroxynitrite [19], cytokines (TNF-α) and pro-inflammatory agents PAF (platelet-activating factor) and leukotriene B_4_ [6], [7], [8]. Secondary stimuli such as reactive oxygen species (ROS) are required to activate primed leukocytes [6], [20]. Upon activation, leukocytes release nitric oxide (NO), ROS and cytokines [7], [18], [21]. In literature, there is no clear evidence that leukocyte recruitment, priming and activation are sequential. There are multiple sources of ROS in blood vessel. ROS from endothelium may act as a secondary stimuli and activate primed leukocytes.

It has been reported that excess NO and O_2_^•−^ production in the vasculature from leukocyte-endothelial cell interactions causes significant increase in peroxynitrite formation as indicated by increased tyrosine nitration [22]. Therefore, together with NO and O_2_^•−^, the formation of peroxynitrite are important contributors for the vascular disorders [4], [18]. The release of NO and O_2_^•−^ by leukocytes increases O_2_^•−^ and peroxynitrite concentration within different regions of the microvasculature [23], [24] and increases endothelial cell Ca^2+^ levels [6], [25]. The increase in endothelial cell Ca^2+^ levels initiates signaling pathways for increasing vascular permeability [25]. Increased vascular permeability causes extravasation of leukocytes into the tissue region leading to tissue injury and complications such as tissue edema [7], [26].

Many studies have investigated the effects of leukocyte-endothelial cell interactions on microvascular functions including permeability [5], [8], [25], vascular tone [27],vessel hemodynamics [28], [29], tissue injury [10], [30] and organ dysfunction [27]. Majority of these studies focused on venular microcirculation. However, there is increasing evidence of leukocyte-endothelial cells interaction in arteriolar microcirculation [5], [15], [16], [17], [26], [29]. The expression of adhesion molecules is upregulated in arteriolar endothelium under inflammatory conditions [5], [15], [16], [29] similar to venules. The leukocyte-endothelial cell interactions between venules and arterioles varies in terms of the adhesion molecules expression levels and their contribution to leukocyte-endothelial interaction and number of leukocytes adhering along the endothelium [15], [17]. Besides the changes in vessel parameters and surrounding cells in arterioles and venules, RBCs in venules are relatively unbound to O_2_ whereas RBC are bound to oxygen in arterioles [31]. Though a significant number of leukocytes can adhere to the venular endothelium and eventually transmigrate [17], a small number of leukocytes is reported to interact with the arteriolar endothelium [15], [17].

Despite the minimal interactions of leukocyte-endothelial cell in arterioles, the vascular disorders in arteriolar microcirculation are similar to those observed in venules [5], [15], [17]. Okamoto et al. [32] reported an increased inflammation in the adventitial region of coronary arterioles that is attributed to increased expression of adhesion molecules in the endothelium, leukocyte infiltration into the adventitia and release of ROS by the leukocytes. Murohara et al. [33] reported that H_2_O_2_ (a ROS) treated arteries showed significant increase in vasoconstriction and adhesion of leukocytes to the endothelium. In addition, Suamgin et al. [5] reported an increase in vessel permeability on transition from control to inflammatory conditions for arterioles and venules. Studies have also shown that increased leukocyte-endothelial cell interactions can lead to increased O_2_^•−^ production from the leukocytes [8] and the endothelium [9]. The increase in O_2_^•−^ production from the leukocytes and endothelium lowers the bioavailability of NO by converting it to peroxynitrite [18], [22]. These studies suggests the possible role that leukocyte-endothelial cell interaction in the vessel NO and peroxynitrite levels can also contribute to vascular disorders in the arterial side of microcirculation. Thus, it is important to understand the transport and interactions of NO, O_2_^•−^ and peroxynitrite in the microcirculation during leukocyte-endothelial cell interaction.

In this study, we examined the biochemical aspects of oxidative stress distribution during the presence of leukocytes (both inactive and activated states) along the endothelium to understand the effects of leukocyte-endothelium interaction on NO, O_2_^•−^ and peroxynitrite profiles. A computational model simulating the biotransport of these species in an arteriolar vessel with leukocytes positioned along the luminal surface of the endothelium was developed in both inactive and active state under several endothelial oxidative stress states. Additionally, the effect of the anti-oxidant superoxide dismutase on the arteriolar NO, O_2_^•−^ and peroxynitrite concentration distribution was also analyzed.

## Materials and Methods

A computational model representing the transport of NO, O_2_^•−^ and peroxynitrite in an arteriole of 50 µm diameter and 500 µm length was developed in this study. Three elliptical leukocytes were positioned along the luminal side of the endothelium. We were interested in understanding the effects of the presence of these leukocytes on the local oxidative and nitrosative stress distribution in the vessel. The model simulations predicted the NO, O_2_^•−^ and peroxynitrite concentration at different regions of the arteriole and within these leukocytes.

### Model Geometry

**Figure 2:**
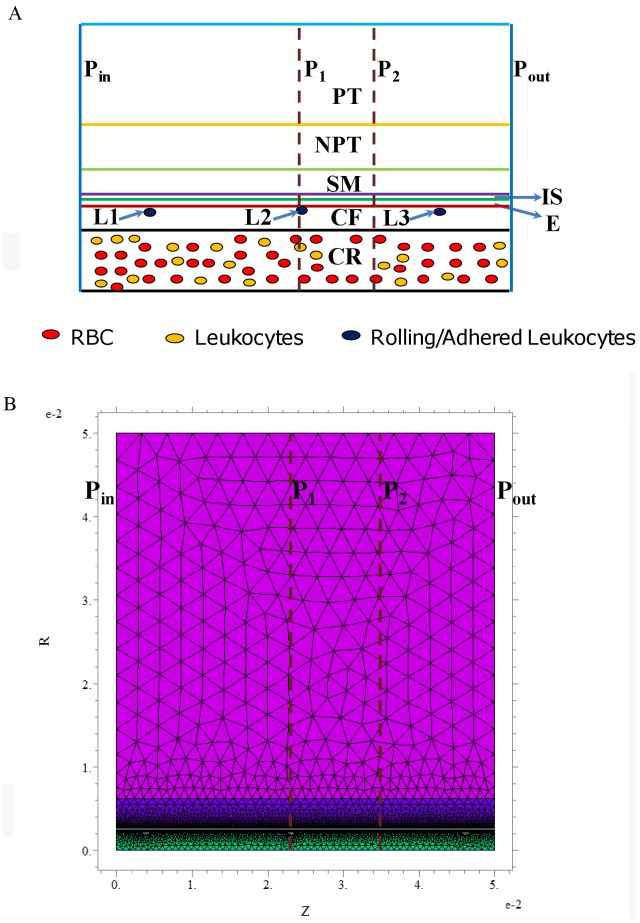
Geometrical description of the problem. **Panel A** shows the schematic of the arteriolar geometry. The geometry consists of concentric cylinders representing the different regions of the arteriole. The different regions fall under the category of either luminal or abluminal region. The luminal and abluminal regions are separated by the endothelial region (E). The luminal region consists of the RBC rich core (CR) and RBC free plasma region (CF). The abluminal region consists of the interstitial region (IS), smooth muscle region (SM), non-perfused (NPT) and capillary perfused (PT) parenchymal regions. L1, L2 and L3 represent the leukocytes interacting with the endothelium. P_in_ and P_out_ represent the inlet and outlet of the arteriolar/vessel segment, respectively. P_1_ and P_2_ represent the locations where the radial concentration profiles of NO, O_2_^•−^ and peroxynitrite were obtained and are located at distances of 230 and 345 µm, respectively from P_in_. **Panel B** shows the schematic of finite element mesh grid with relative accuracy set to 0.001.

A cylindrical geometry with concentric cylinders was used to represent the arteriole and its associated regions as shown in Figure 2. These regions include the luminal RBC (red blood cell) rich region (CR), RBC free region next to the vessel wall (CF), endothelium (E), interstitial space (IS) between the endothelium and smooth muscle cells, smooth muscle layer (SM), non-perfused parenchymal tissue (NPT) and perfused parenchymal tissue (PT) region. The CR region in the lumen of the arteriole was considered to have a homogenous solution of RBC’s [34]. The thickness of these different regions is shown in Table 1. Three leukocytes were positioned on the luminal side of the endothelium for all the cases simulated and were named as L1, L2 and L3, respectively. Details about the leukocyte geometry and positioning of the leukocytes are described in the “Model Parameters” subsection.

**Table 1:** Model Parameters.

Parameter	Value	Units	Reference
Systemic hematocrit	45	%	[34], [41]
Capillary hematocrit	30	%	[40], [41]
C_RBC-Hb_	20.3	mM	[40], [41]
Arteriole radius	25	µm	Text
CF region thickness	4.5	µm	[35], [40], [41]
Endothelial thickness	0.5	µm	[40], [41]
IS region thickness	0.5	µm	[41]
SM region thickness	6	µm	[40], [41]
NPT region thickness	30	µm	[40], [41]
O_2_ concentration	27	µM	[41]
CO_2_ concentration	1.1	mM	[41]
SOD concentration	1, 10	µM	[56], Text
P_NO,E_	5.3×10^−12^	moles.cm^−2^.s^−1^	[40], [43], [44]
P_O2_^•−^_,E_	2.7×10^−13^, 1.06×10^−12^, 5.3×10^−12^	moles.cm^−2^.s^−1^	[41], Text
P_NO,PT_	8.6×10^−7^	M.s^−1^	[40], Text
P_O2_^•−^_,PT_	4.3×10^−8^, 1.72×10^−7^, 8.6×10^−7^	M.s^−1^	[41], Text
P_NO,L1,L2,L3_	10	µM.s^−1^	[45]
P_O2_^•−^_,L1,L2,L3_	4.11×10^−5^	M.s^−1^	[46]
D_NO_	3.3×10^−5^	cm^2^.s^−1^	[41]
D_O2_^•−^	2.8×10^−5^	cm^2^.s^−1^	[41], [50]
D_Per_	2.6×10^−5^	cm^2^.s^−1^	[41], [50]
f (tissue)	0.640		[41]
f (lumen)	0.817		[41]
v_zmax_	0.5	cm.s^−1^	[36], [40]
k_NO-RBC_	0.2×10^5^	M^−1^.s^−1^	[34], [55]
k_cap_	1.77	s^−1^	[40], [41]
k_NO-O2_	9.6×10^6^	M^−2^.s^−1^	[40], [41]
k_SM_	5×10^4^	M^−1^.s^−1^	[41]
k_Per_	6.7×10^9^	M^−1^.s^−1^	[41], [89]
k_sod_	1.6×10^9^	M^−1^.s^−1^	[41], [89]
k_oxy_	4.5	s^−1^	[56]
k_CO2_	5.6×10^4^	M^−1^.s^−1^	[41]
k_hb1_	30	s^−1^	[56]
k_NO-Per_	9.1×10^4^	M^−1^.s^−1^	[41]

### Model Assumptions

The following were the assumption of the mathematical model:

1. The convective transport of the species (NO, O_2_^•−^ and peroxynitrite) was assumed in the lumen (CR and CF regions) due to blood flow.
2. The geometric parameters including thickness of the CR and CF regions were based on systemic hematocrit and vessel diameter [35].
3. The NO production rate from the endothelium was proportional to the shear stress exerted on the vessel wall due to blood flow [36]. The shear stress on the vessel wall was calculated from apparent viscosity of blood (3 cPs), vessel diameter and physiological blood velocity (0.5 cm.s^−1^) using Newton’s law of viscosity [36]. The apparent blood viscosity was obtained from literature and is a function of systemic hematocrit [37].
4. The basal case O_2_^•−^ production in the endothelium and capillary was assumed at 5% of their respective NO production. In basal case, leukocytes in inactivated state were modeled. We changed the NO production rate from the leukocytes and the O_2_^•−^ production rate in the endothelium, capillary and leukocytes depending on simulated-cases. These changes are described later in this section.
5. The boundaries separating the different regions of the arteriole geometry and the outer surface of the perfused parenchymal tissue (PT) region were considered rigid.
6. The simulations were performed at steady state.
7. We assumed laminar flow in the lumen. The presence of leukocytes may affect the blood velocity profile. However, at physiological velocity, the convective transport of the modeled species can be neglected [38], [39].
8. The arteriole geometry was considered to be axially symmetric and hence the computational domain involved only half of the complete vessel geometry starting from the central axis of the vessel to the outer surface of PT region. This assumption will affect the profile only when there is another leukocyte in the vicinity.
9. We used three leukocytes for our simulations based on the experimental observations for leukocyte rolling flux and rolling velocity for TNF-α treated arterioles of ICAM-1 KO mice and wild type (WT) mice [15]. Their reported values were 7 leukocytes/40 s and 32.2 µm.s^−1^ for ICAM-1 KO mice and 13 leukocytes/40 s and 53.5 µm.s^−1^ for WT mice. These two values results in 3.2 and 3.03 leukocytes per 500 µm vessel length. The calculations were performed based on the time taken by one leukocyte to traverse the length of the arteriole and the leukocyte rolling flux in that time period. It was also reported that in inflammatory conditions about 0.64 leukocytes/100 µm were adhered to arteriolar endothelium [15], which translated to 3 adhered leukocytes for the vessel length used in this study (500 µm).

### Governing Equations

The transport of NO, O_2_^•−^ and peroxynitrite across different regions of the arteriole and within the leukocytes occurs due to both diffusion and convection. Additionally, these three species are also produced and consumed in different regions of the arteriole. Thus, the governing equation for their transport is a combination of the diffusion, convection, production and reaction kinetic terms. The transport of the three different chemical species (NO, O_2_^•−^ and peroxynitrite) were represented by three separate equations. The equations for mass transfer of NO, O_2_^•−^ and peroxynitrite across all the regions of the arteriole and the leukocyte were in the following form:
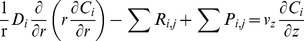
(1)Where the suffix i represents the chemical species (NO, O_2_^•−^ and peroxynitrite) and j the respective regions (i.e. CR, CF, E, IS, SM, NPT, PT, L1, L2 and L3). D_i_ (cm^2^.s^−1^) is the diffusivity of species i, C_i_ (nM) is the concentration of species i, R_i,j_ (M.s^−1^) represents the reaction rate of species i in the region j and P_i,j_ represents the production rate of species i in the region j (M.s^−1^). v_z_ (m.s^−1^) is the axial component of blood flow velocity and is a function of radial distance r [40]. The blood flow was within the laminar flow range based on apparent Reynolds number calculations using a value of 3 cPs for apparent blood viscosity [36]. Therefore, a parabolic velocity distribution of luminal blood flow was considered in this study. The velocity distribution was represented as a function of radial distance (r) in the following form [40]:
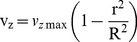
(2)Where R (µm) is the radius of the arteriole (25 µm) and vzmax is the velocity of blood at the center of the arteriole (r = 0), which is also the maximum value of velocity.

### Boundary Conditions

The zero flux boundary condition was applied to the center of the arteriole (r = 0) and at the outer surface of the perfused parenchymal tissue (PT) region. The boundary conditions of continuity of NO, O_2_^•−^ and peroxynitrite were applied at all the interfaces with the exception of the interface between (a) the endothelium and the luminal cell free (CF) region and (b) the interstitial space (IS) and endothelium (E). At these interfaces, the boundary conditions involve the endothelium based NO and O_2_^•−^ production. Mathematically, the boundary condition at the interface between cell free (CF) and endothelium (E) region can be represented as follows:
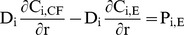
(3)The boundary condition at the interface between the interstitial space (IS) and endothelium (E) can be mathematically represented as:
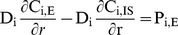
(4)In equation (3) and equation (4) the term Pi,E represents NO and O_2_^•−^ production in the endothelium. In the case of peroxynitrite, the continuous boundary condition was applied across all the interfaces as it was not produced directly in any region of the arteriole or within the leukocytes (PPer, j = 0).

### Cases Simulated

Our understanding of the effects of the presence of leukocytes along the endothelium under different levels of endothelial oxidative stress and during activation of leukocytes is lacking. Five different cases were simulated to mimic the different levels of endothelial oxidative stress and leukocyte activation. The cases are as follows:

#### Case 1

A normal physiologic condition − In these simulations, basal level of endothelial oxidative stress (O_2_^•−^ production in the endothelium and capillary was set at 5% of their respective NO production) and leukocytes in inactivated state were modeled.

#### Case 2

Endothelial oxidative stress condition − In these simulations, the O_2_^•−^ production in the endothelium and capillary was set at 20% of their respective NO production and leukocytes were in inactivated state.

#### Case 3

Combination of endothelial oxidative stress and activation of leukocytes − The O_2_^•−^ production in the endothelium and capillary was set at 20% of their respective NO production and leukocytes were in activated state producing NO and O_2_^•−^. The next two cases are based on this case. One is a therapeutic scenario and another is a severe oxidative stress condition that is the exacerbation of the Case 3 in the absence of any treatment.

#### Case 4

Combination of endothelial oxidative stress, activated leukocytes and increased SOD concentration (a therapeutic scenario) − SOD concentration was increased by an order of magnitude from 1 µM to 10 µM globally across all regions of the arteriole geometry. Other conditions were the same as the Case 3.

#### Case 5

Severe oxidative stress condition − The O_2_^•−^ production in the endothelium and capillary was set at 100% of their respective NO production and leukocytes were in activated state producing NO and O_2_^•−^.

### Sources of NO and O_2_^•−^ Production

NO and O_2_^•−^ production occurs at the luminal and abluminal surfaces of the endothelium [41]. The primary endothelium based NO source is the enzyme endothelial nitric oxide synthase (eNOS) while the primary endothelial O_2_^•−^ sources include NADPH oxidase and uncoupled eNOS [41], [42]. NO and O_2_^•−^ production also takes place in the perfused section of the parenchymal tissue region (PT) from capillary endothelium [34], [41] and in the leukocytes upon activation [7], [20], [25]. Kavdia and Popel [36] proposed the following relationship between wall shear stress and endothelial NO release rate:
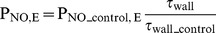
(5)P_NO_control,E_ is the basal rate of NO release by the endothelium at control wall shear stress (τ_wall_control_) of 24 dyne.cm^−2^ corresponding to the physiological blood velocity of 0.5 cm.s^−1^ and an apparent blood viscosity of 3 cPs in a 50 µm diameter arteriole [36]. The control NO production rate in the endothelial region (P_NO_control,E_) used in this study was 5.3×10^−12^ moles.cm^−2^.s^−1^ based on the experimental data of Malinski et al. [43], [44] in rabbit aorta. The basal rate of capillary based NO production in PT region is calculated from the endothelium based NO release rate by Kavdia and co-workers [34], [35] and was estimated to be (P_NO_control,PT_) 8.6×10^−7^ M.s^−1^.

The O_2_^•−^ production in these two regions (E and PT) was assumed to be a fraction of their respective NO production rates [41] and varied as described in Cases Simulated subsection above. The leukocyte NO and O_2_^•−^ production rate were assumed to be 10 µM.s^−1^ and 41.1 µM.s^−1^, respectively. The leukocyte NO production rate was calculated from the inducible NOS (iNOS) enzyme based NO production rates from leukocytes of 3 pmoles.s^−1^/10^6^ cells reported by Nalwaya and Deen [45] The leukocyte O_2_^•−^ production rate was calculated from the O_2_^•−^ release rates of 12.33 pmoles.s^−1^/10^6^ cells reported by Tsukimori et al. [46]. The volume of 300 µm^3^ for a single leukocyte reported by Ting-Beall et al. [47] was used to convert both these production rates into appropriate Molar units. The values of the NO and O_2_^•−^ production rates in the endothelium, perfused parenchymal region and leukocytes for the different cases simulated (Case 1 to Case 5) are shown in Table 2.

**Table 2:** Endothelial, capillary and leukocyte based NO and O_2_^•−^ production rates and SOD concentration for the different cases simulated.

Case Number	P_i,E_ (pmoles.cm^−2^.s^−1^)		P_i,PT_ (µM.s^−1^)		C_SOD_ (µM)	P_i,L_ 1 (µM.s^−1^)	
	NO	O_2_^•−^	NO	O_2_^•−^		NO	O_2_^•−^
Case 1	5.3	0.27	0.86	0.043	1	0	0
Case 2	5.3	1.06	0.86	0.172	1	0	0
Case 3	5.3	1.06	0.86	0.172	1	10.0	41.1
Case 4	5.3	1.06	0.86	0.172	10	10.0	41.1
Case 5	5.3	5.3	0.86	0.86	1	10.0	41.1

1 **P_i,L_** refers to the production rate of NO and O_2_^•−^ from the leukocytes L1, L2 and L3.

### Chemical Reactions

NO, O_2_^•−^ and peroxynitrite undergo chemical reactions at all or specific regions within the arteriole geometry. The specifics of these reactions are shown in Table 3. The rate constants pertaining to the different chemical reactions were either directly procured from literature or calculated (such as k_cap_). Amongst the reactions shown in Table 3, the reaction involving NO and hemoglobin (Hb) in the CR and PT regions involve the two reactants (NO and Hb) and the RBC. The rate of consumption of NO by Hb in the CR region is represented as the product of NO reaction rate constant with RBC Hb (k_NO-RBC_), concentration of Hb in a single RBC (C_RBC-Hb_), systemic hematocrit and NO concentration. The values of these parameters and their sources are shown in Table 1. In the PT region, the rate of reaction between NO and RBC is represented as the product of effective rate constant of reaction between NO and Hb in blood flowing in capillaries perfusing in the PT region (k_cap_) and the concentration of NO. k_cap_ is obtained by multiplying the NO reaction rate constant with RBC Hb (k_NO-RBC_), concentration of Hb in RBC (C_RBC-Hb_), capillary hematocrit and fractional capillary volume. The value of fractional capillary volume used was 0.0146 based on experimental data reported for hamster retractor muscle [35], [48]. The other parameters used in calculation of k_cap_ are shown in Table 1. Details regarding the calculation of k_cap_ can be found in articles by Kavdia and co-workers [35], [40], [41].

**Table 3:** Description of reactions and their kinetic expressions used in the model.

Rxn #	Reaction	Regions	Mathematical Expression	Ref.
Rxn 1	NO and Hb	CR	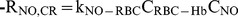	[40], [41]
Rxn 2	NO and Hb	PT	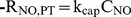	[40], [41]
Rxn 3	NO and O_2_	CF, E, IS & NPT	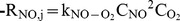	[41]
Rxn 4	NO and sGC	SM	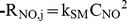	[40], [41]
Rxn 5	NO and O_2_^•−^	All	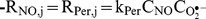	[41], [56]
Rxn 6	O_2_^•−^ and SOD	All	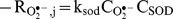	[41], [56]
Rxn 7	Peroxynitrite Dissociation	All	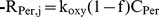 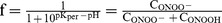	[41], [56]
Rxn 8	Peroxynitrite & CO_2_	All	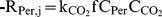	[41]
Rxn 9	Peroxynitrite & Hb	CR & PT	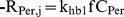	[56]
Rxn 10	Peroxynitrite & NO	All	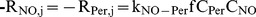	[41], [56]

### Model Parameters

The model parameters are reported in Table 1. The dimensions of the elliptical leukocyte (semi-major and semi-minor axis) were assumed to be 4.9 and 3.5 µm, respectively. The leukocyte dimensions were based on the observations of deformation index of 1.36 by Damiano et al. [49] and a spherical diameter of leukocytes in resting state of 8.3 µm by Ting-Beall et al. [47]. Damiano et al. [49] observed that adhesion molecule expression and shear stress due to blood flow contribute to change in leukocyte morphology from circular to elliptical and expressed this change in terms of a deformation index. The centers of these three leukocytes were located at distances of 38, 230.8 and 461.5 µm, respectively from the vessel inlet (P_in_) and at a radial distance of 3.5 µm from the luminal surface of the endothelium as shown in Figure 2A. The justification for other geometrical parameters is presented in earlier studies [35], [40], [41]. For all the regions of the arteriole and within the leukocytes, the diffusivity of NO, O_2_^•−^ and peroxynitrite were 3.3×10^−5^, 2.8×10^−5^ and 2.6×10^−5^ cm^2^.s^−1^, respectively [41], [50].

### Numerical Solution

On the basis of Equation 1, a system of partial differential equations were generated for representing the transport and reactions involving NO, O_2_^•−^ and peroxynitrite. These equations with the appropriate boundary conditions were solved numerically using the finite element software package FlexPDE 5.0 (PDE solutions Inc., Antioch, CA, USA). This software uses adaptive meshing that generates finer elements in regions with steep concentration gradient and coarse meshing elsewhere within the domain as shown in Figure 2B. This optimizes the computational resources required for the simulations. For all the simulations used in this study, we used a relative accuracy of 0.001.

## Results

The model used in this study predicts the steady state NO, O_2_^•−^ and peroxynitrite concentration distribution and radial profiles under normal and oxidative stress conditions in the presence of three leukocytes located along the luminal surface of the endothelium. The steady state radial concentration profiles of NO, O_2_^•−^ and peroxynitrite were obtained at two distinct locations along the axial direction of the arteriole as shown in Figure 2A and B. The first location passes through one of the leukocyte (location P_1_ in Figure 2A and B) present along the endothelium (L2). The second location passes through the midpoint between the centers of two leukocytes present along the endothelium (location P_2_ in Figure 2A and B between leukocytes L2 and L3). The first location was at a distance of 230 µm and the second at a distance of 345 µm. The NO concentration increased radially along the arteriole starting at the vessel center. The O_2_^•−^ and peroxynitrite concentration increased radially starting at the vessel center to a maximum at the endothelium (in some cases near the endothelium as stated) and reduced thereafter. The maximum endothelial NO, O_2_^•−^ and peroxynitrite concentrations at locations P_1_ and P_2_ for all the cases (Case 1−5) are shown in Table 4. The NO, O_2_^•−^ and peroxynitrite concentration range in the different regions of the arteriole is shown in Table 5.

**Table 4:** Maximum endothelial concentrations of NO, O_2_^•−^ and peroxynitrite at location P_1_ 2 and location P_2_ 3. C_Per_ refers to the peroxynitrite concentration.

Case Number	C_NO_ (nM)		C_O2_^•-^ (pM)		C_Per_ (nM)	
	P_1_	P_2_	P_1_	P_2_	P_1_	P_2_
Case 1	262	250	431	426	2	2
Case 2	240	229	1761	1741	6	6
Case 3	226	229	5801	1741	41	6
Case 4	285	251	1064	704	8	1
Case 5	153	150	13447	9591	51	24

2 The location P_1_ refers to a straight line passing through the leukocyte L2 at a distance of 230 µm from the vessel inlet P_in_ as seen in Figure 2.

3 The location P_2_ refers to a straight line passing the centers of leukocytes L2 and L3 at a distance of 345 µm from the vessel inlet P_in_ as seen in Figure 2.

**Table 5:** Concentration range of NO, O_2_^•−^ and peroxynitrite at different regions of the arteriole geometry.

Case Number	Regions	C_NO_ (nM)	C_Per_ (nM)	C_O2_^•−^ (pM)
**Case 1**	**CR**	0–140	0–1	0–7
	**CF**	3–251	0.1–2	3–310
	**E**	6–262	1–2	251–500
	**IS**	69–264	1–2	247–500
	**SM**	134–292	0.4–2	0.4–290
	**NPT**	210–389	0.1–1	0–6
	**PT**	375–469	0.4–1	7–10
**Case 2**	**CR**	0–130	0–4	0–28
	**CF**	0.2–230	0.4–6	14–1270
	**E**	2–241	1–6	1040–2040
	**IS**	65–242	1–6	1020–2030
	**SM**	86–266	1–6	2–1240
	**NPT**	192–353	1–3	0–26
	**PT**	340–423	1–3	30–45
**Case 3**	**CR**	0–119	0–33	0–7470
	**CF**	3–219	0–44	16–6430
	**E**	4–229	1–44	1090–6830
	**IS**	64–232	1–41	1044–5063
	**SM**	88–260	2–37	2–2800
	**NPT**	191–351	1–12	0–26
	**PT**	340–423	1–3	29–45
**Case 4**	**CR**	0–162	0–8	0–990
	**CF**	2–274	0–9	0–1100
	**E**	0.4–286	0.1–9	220–1100
	**IS**	61–287	0.2–8	210–1000
	**SM**	95–305	0.3–7	0–250
	**NPT**	209–393	0.1–2	0–5
	**PT**	375–469	0.4–1	7–10
**Case 5**	**CR**	0–80	0–43	0–8200
	**CF**	0.2–145	0.2–53	20–12600
	**E**	0–152	0–53	6200–13600
	**IS**	45–152	4–51	6100–13600
	**SM**	61–164	6–48	20–8000
	**NPT**	121–201	2–18	0–170
	**PT**	206–241	6–10	220–290

### Concentration Distribution and Profiles of NO, O_2_^•−^ and Peroxynitrite Under Normal Physiological Conditions (Case 1)

Under normal physiological conditions, a small number of leukocytes may still roll along the endothelium primarily acting as surveillance against antigens or pathogenic foreign bodies [51]. Additionally, a small amount of O_2_^•−^ is released from endothelial cells under normal physiologic conditions [41], [52]. In this case (Case 1), leukocytes were considered to be inactivated as they are neither primed nor activated under normal physiologic conditions [7], [20], [53], [54].

**Figure 3:**
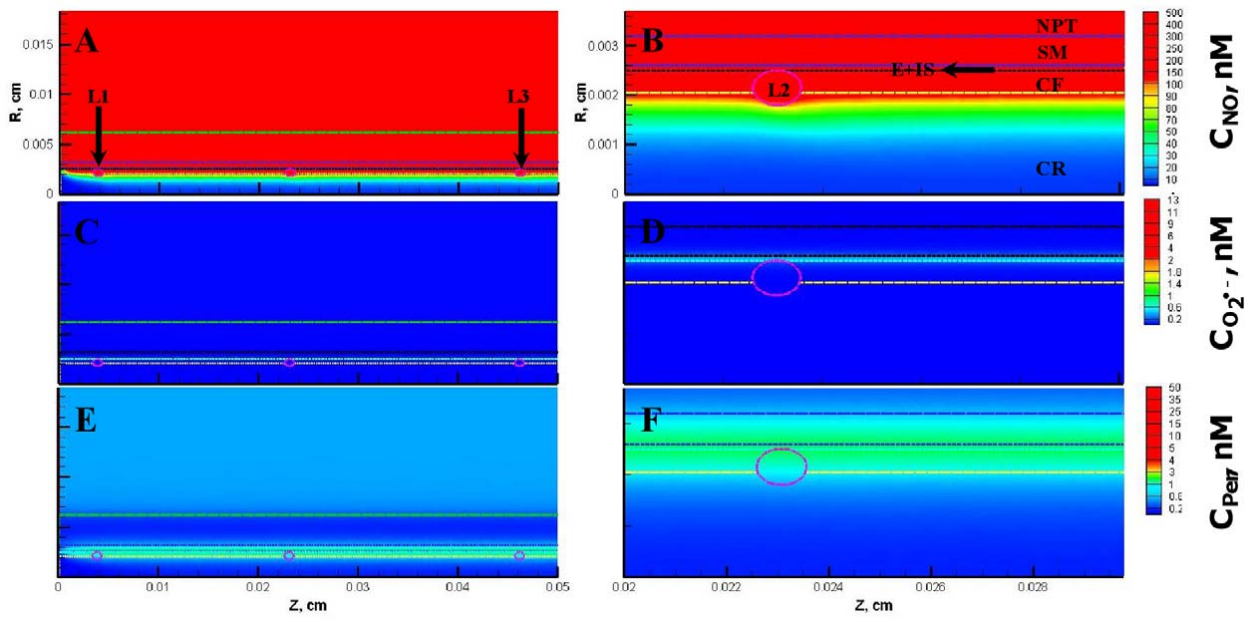
Concentration distribution under normal physiological conditions (Case 1). **Panel A, C and E** shows the NO, O_2_^•−^ and peroxynitrite (referred as C_Per_) concentration distribution, respectively across the entire arteriolar geometry. **Panel B,** **D** and **F** shows the NO, O_2_^•−^ and peroxynitrite concentration distribution, respectively across a segment of the arteriolar geometry between 200 and 300 µm encompassing the luminal, E, SM and NPT regions. The endothelial and capillary based O_2_^•−^ production rates in this case were 5% of their respective NO production rates and the leukocytes were considered inactive.

**Figure 4:**
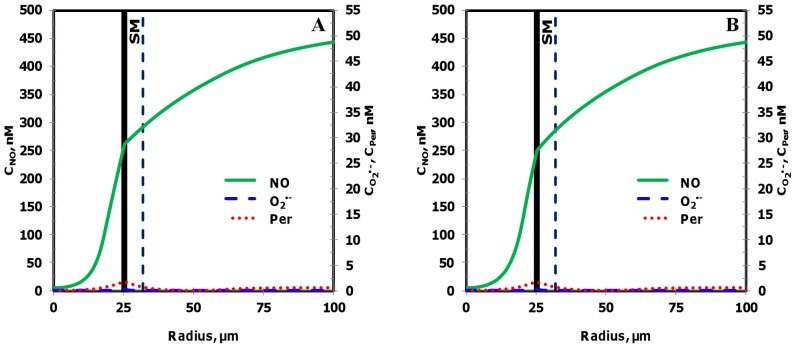
Radial concentration profiles at locations P_1_ and P_2_ for the Case 1. **Panel A** and **B** shows the radial concentration profiles of NO, O_2_^•−^ and peroxynitrite at the location P_1_ and P_2_, respectively.

The NO, O_2_^•−^ and peroxynitrite concentrations are shown in Figure 3A, C and E, respectively for the entire vessel and in Figure 3B, D and F, respectively for a segment of the vessel between 200 and 300 µm encompassing the luminal, endothelial (E), smooth muscle (SM) and non-perfused parenchymal tissue (NPT) region. The concentration range of NO, O_2_^•−^ and peroxynitrite within the leukocytes were 88−248 nM, 0.2−200 pM, and 1−2 nM, respectively. The concentration range of NO, O_2_^•−^ and peroxynitrite at other regions of the arteriole are shown in Table 5. The radial concentration profiles for NO, O_2_^•−^ and peroxynitrite at location P_1_ and P_2_ are shown in Figure 4 A and B, respectively and their maximum values at the endothelium are shown in Table 4.

### Concentration Distribution and Profiles of NO, O_2_^•−^ and Peroxynitrite Under The Endothelial Oxidative Stress Conditions (Case 2)

**Figure 5:**
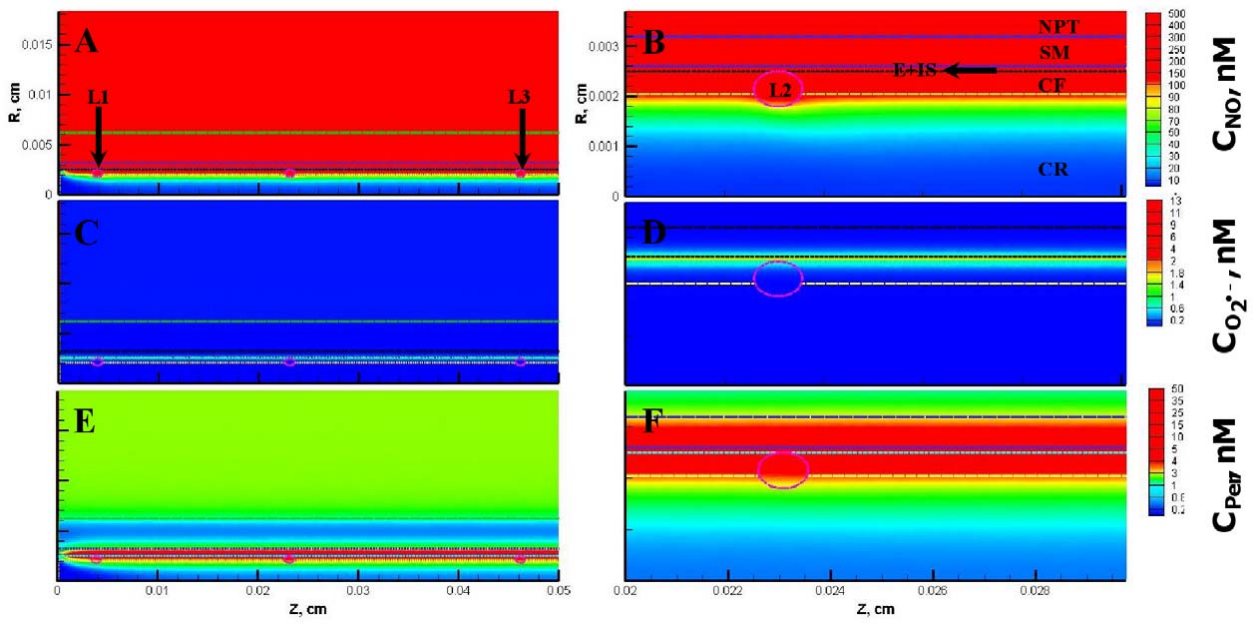
Concentration distribution under conditions of endothelial oxidative stress (Case 2). The NO, O_2_^•−^ and peroxynitrite concentration distribution are shown for the entire arteriolar geometry in **Panels A, C, and E**, respectively and across the 200–300 µm region in **Panels B, D and F**, respectively. The endothelial and capillary based O_2_^•−^ production rates in this case were 20% of their respective NO production rates and the leukocytes were considered inactive.

Under the endothelial oxidative stress condition (Case 2), the NO, O_2_^•−^ and peroxynitrite concentrations are shown in Figure 5A, C and E, respectively for the entire vessel and in Figure 5B, D and F, respectively for a segment of the vessel between 200 and 300 µm. Compared to the Case 1, the maximum concentration of NO decreased at all regions of the blood vessel and in the leukocytes. The concentration range of NO, O_2_^•−^ and peroxynitrite within the leukocytes were 81−231 nM, 1−1022 pM, and 2−6 nM, respectively. In comparison to the Case 1, the maximum NO concentration changed by 0.9 fold, whereas the maximum O_2_^•−^ and peroxynitrite concentrations increased by 5 and 4 fold, respectively. In the CR, CF, E and SM regions, the maximum NO concentration changed by 0.9 fold while the maximum O_2_^•−^ and peroxynitrite concentration increased by 4 fold, respectively.

**Figure 6:**
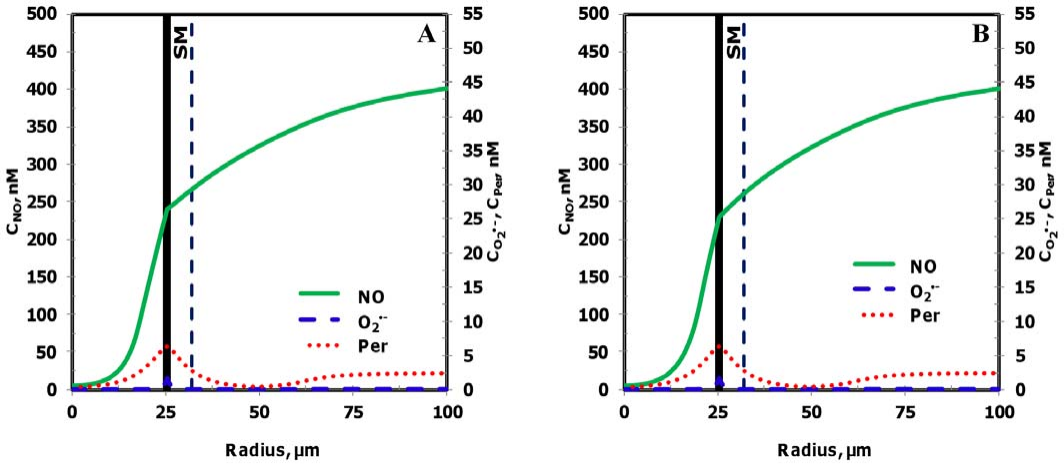
Radial concentration profiles at locations P_1_ and P_2_ for the Case 2. **Panel A** and **B** shows the radial concentration profiles of NO, O_2_^•−^ and peroxynitrite at the location P_1_ and P_2_, respectively.

The radial concentration profiles of NO, O_2_^•−^ and peroxynitrite at location P_1_ and P_2_ are shown in Figure 6 A and B, respectively. In comparison to the Case 1, the maximum endothelial NO concentration changed by 0.9 fold whereas the maximum endothelial O_2_^•−^ and peroxynitrite concentration increased by 4 fold, at location P_1_ and P_2_, respectively (see Table 4).

### The Combined Endothelial Oxidative Stress Conditions and the Activation of Leukocytes (Case 3): Significant Increase in Peroxynitrite Concentration Over a Length of 100 µm in Vicinity of a Leukocyte

**Figure 7:**
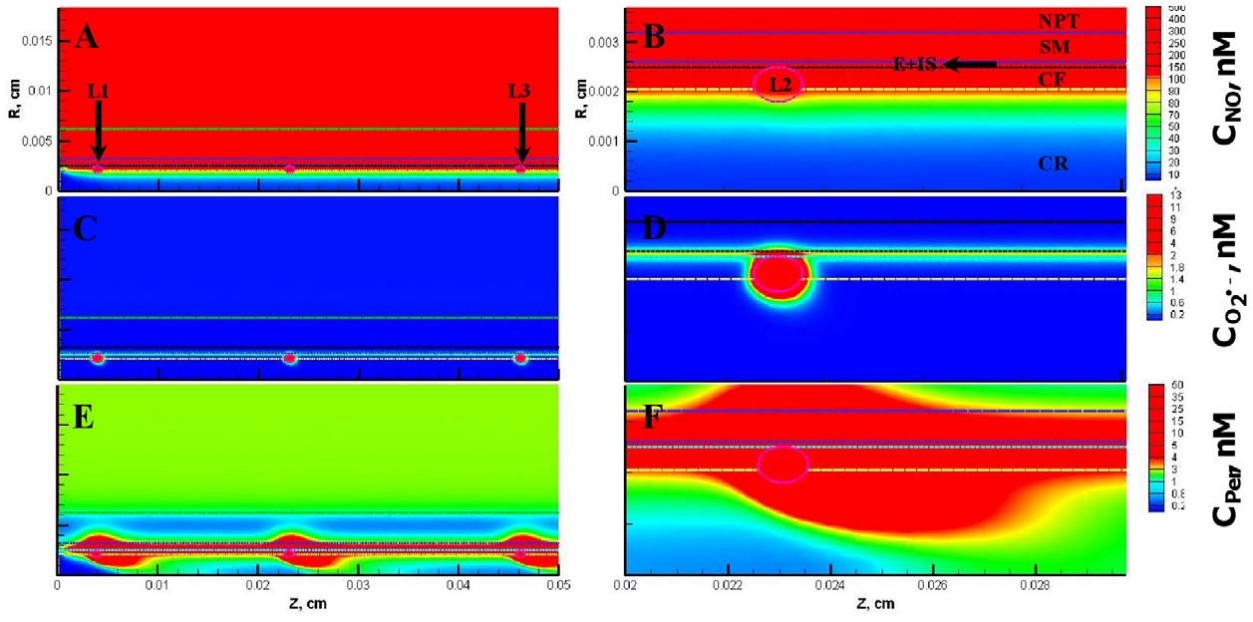
Concentration distribution under combination of endothelial oxidative stress and activation of leukocytes (Case 3). The NO, O_2_^•−^ and peroxynitrite concentration distribution are shown for the entire arteriolar geometry in **Panels A, C, and E,** respectively and across the 200–300 µm region in **Panels B, D and F,** respectively. The O_2_^•−^ production in the endothelium and capillary in this case were 20% of their respective NO production and the leukocytes were in activated state producing NO and O_2_^•−^.

For the combination of the endothelial oxidative stress condition with the activation of leukocytes (Case 3), the NO, O_2_^•−^ and peroxynitrite concentration distribution are shown in Figure 7. Compared to the Case 2, the maximum NO concentration decreased at all regions of the blood vessel excluding PT region (remained unchanged in PT region) and the leukocytes. The concentration range of NO, O_2_^•−^ and peroxynitrite within the leukocytes were 78−216 nM, 7000−14000 pM and 23−53 nM, respectively. In comparison to the Case 2, the maximum NO concentration changed by 0.9 fold, whereas the maximum O_2_^•−^ and peroxynitrite concentrations increased by 14 and 8 fold, respectively. In comparison to the Case 2, the maximum NO concentration changed by 0.9 fold, the maximum O_2_^•−^ concentration increased by 267, 5, 3 and 2 fold, and the maximum peroxynitrite concentration increased by 9, 7, 7 and 6 fold in the CR, CF, E and SM regions, respectively.

At the CF, E and SM regions, the activation of leukocytes with the endothelial oxidative stress conditions results in greater fold increase in peroxynitrite concentration as compared to increase in O_2_^•−^ concentration. This is due to the iNOS related NO production from the activated leukocytes that reacts with O_2_^•−^ to form peroxynitrite. It also shows the ability of peroxynitrite to diffuse greater distances compared to O_2_^•−^ [41]. In the CR region, the increase in O_2_^•−^ concentration is greater than peroxynitrite due to rapid scavenging of both NO and peroxynitrite by hemoglobin [55], [56].

**Figure 8:**
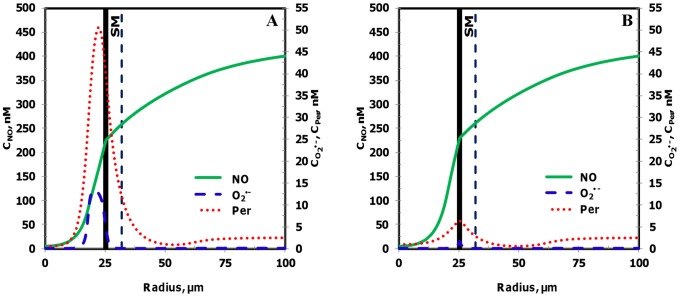
Radial concentration profiles at locations P_1_ and P_2_ for the Case 3. **Panel A** **and B** shows the radial concentration profiles of NO, O_2_^•−^ and peroxynitrite at the location P_1_ and P_2_, respectively.

At the location P_1_ and P_2_, the radial concentration profiles for NO, O_2_^•−^ and peroxynitrite are shown in Figure 8 for the Case 3. The location for the maxima for O_2_^•−^ and peroxynitrite at location P_1_ shifted from the endothelium (as observed in the previous cases (Case 1 and 2)) to the lumen at distances of 20.5 and 22.3 µm, respectively from the center of the vessel. The maximum O_2_^•−^ and peroxynitrite concentrations were 13300 pM and 51 nM, respectively. In comparison to the Case 2, the maximum endothelial NO concentration changed by 0.9 fold and the maximum O_2_^•−^ and peroxynitrite concentration increased by 3 and 6 fold, respectively at the location P_1_ (Table 4). However at the location P_2_, the NO, O_2_^•−^ and peroxynitrite concentrations remained unchanged. Furthermore, the peroxynitrite increased in the vicinity of the activated leukocytes when compared in contour profiles of the Case 2 (Figure 5F) with the Case 3 (Figure 7F). A region of 100 µm in length can experience a significant increase in peroxynitrite concentration upon activation of a single leukocyte.

### SOD Effectively Reduces Oxidative Stress Associated with the Endothelial Region and the Activation of Leukocytes (Case 4)

**Figure 9:**
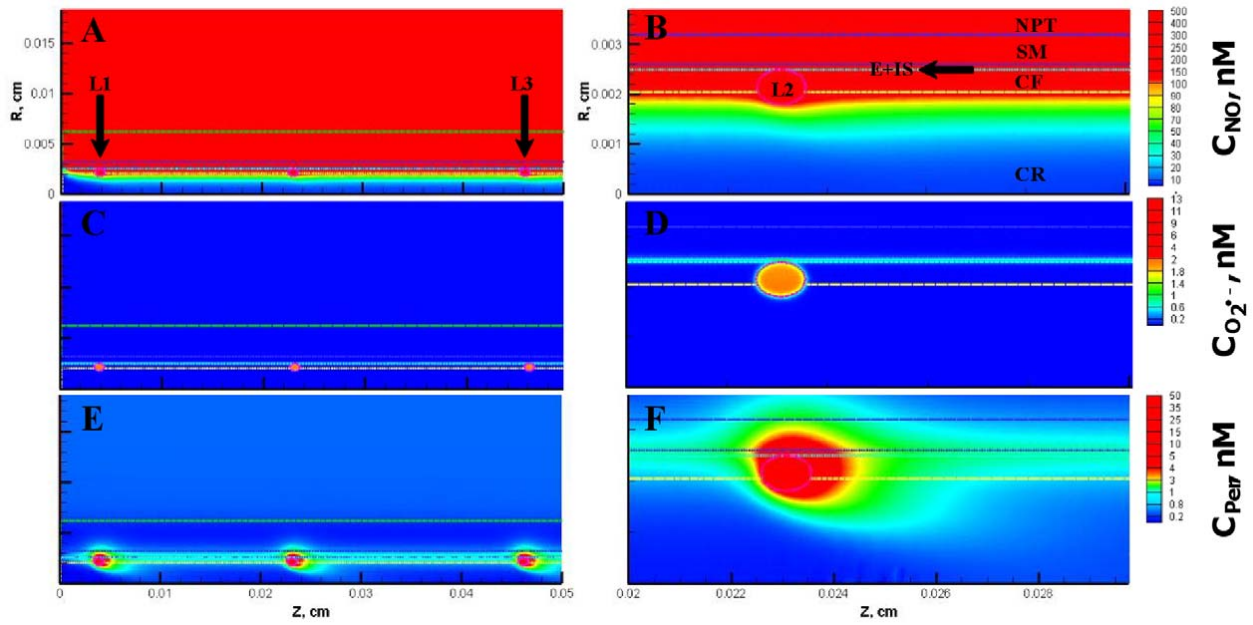
Concentration distribution under endothelial oxidative stress, activated leukocytes and increased SOD concentration (Case 4). The NO, O_2_^•−^ and peroxynitrite concentration distribution are shown for the entire arteriolar geometry in **Panels A, C, and E,** respectively and across the 200–300 µm region in **Panels B, D and F,** respectively. The O_2_^•−^ production in the endothelium and capillary in this case were 20% of their respective NO production and the leukocytes were in activated state producing NO and O_2_^•−^. The SOD concentration across all the regions of the arteriole and within the leukocytes was set at 10 µM.

**Figure 10:**
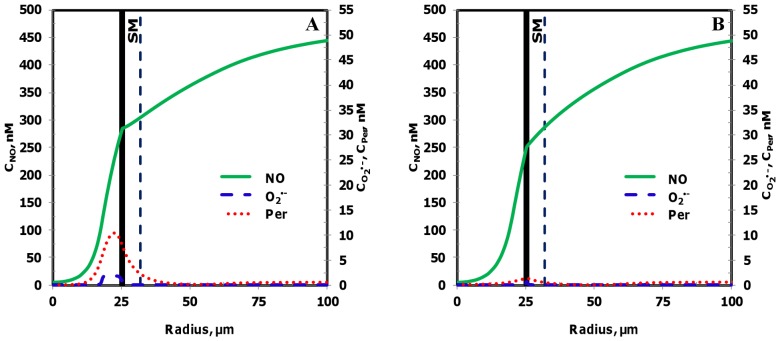
Radial concentration profiles at locations P_1_ and P_2_ for the Case 4. **Panel A** and **B** shows the radial concentration profiles of NO, O_2_^•−^ and peroxynitrite at the location P_1_ and P_2_, respectively.

Experimental studies have demonstrated that the O_2_^•−^ scavenger superoxide dismutase (SOD) can inhibit leukocyte adhesion, reduce adhesion molecule expression and attenuate increase in vascular permeability [8], [25]. It is thus important to understand the effect of increased SOD on the concentration profiles from the Case 3. For the Case 4, we increased the SOD concentration from 1 to 10 µM. Figures 9 and 10 shows the resulting concentration distributions and radial profiles, respectively. In comparison to the Case 3, the maximum O_2_^•−^ and peroxynitrite concentration reduced across all the regions of the blood vessel and in the leukocytes.

Within the leukocytes for the Case 4, the concentration range for NO, O_2_^•−^ and peroxynitrite were 106−277 nM, 1300−1940 pM and 5−11 nM, respectively. The maximum NO concentration increased 1.2 fold while the maximum O_2_^•−^ and peroxynitrite concentrations changed by 0.1 and 0.2 fold, respectively in comparison to the Case 3. In comparison to the Case 3, the maximum NO concentration increased by 1.4, 1.2, 1.2 and 1.1 fold in the CR, CF, E and SM regions, respectively (see Table 5). The maximum O_2_^•−^ and peroxynitrite concentrations changed by 0.08−0.2 fold and by 0.2 fold, respectively in the CR, CF, E and SM regions. The peroxynitrite concentration significantly reduced in the vicinity of the leukocyte as compared to the Case 3.

Furthermore at the locations P_1_ and P_2,_ the maximum endothelial NO concentration increased by 1.2 fold, respectively in comparison to the Case 3. The O_2_^•−^ and peroxynitrite concentration reached their respective maximum values of 1938 pM and 10 nM at distances of 20.3 and 22.3 µm, respectively from the center of the vessel at the location P_1_. The maximum O_2_^•−^ concentrations changed by 0.2 and 0.4 fold and the maximum peroxynitrite concentrations changed by 0.2 fold at the locations P_1_ and P_2_, respectively.

### An Onset of Severe Oxidative Stress Condition Affects All Regions of the Microvasculature (Case 5)

Endothelial oxidative stress in conjunction with activation of leukocytes causes a significant increase in peroxynitrite concentration in the endothelial region. An increased peroxynitrite concentration in the endothelial region can promote the oxidation of the eNOS co-factor BH_4_ to BH_2_ and causes eNOS uncoupling that results in reduction of eNOS based NO production, increased eNOS based O_2_^•−^ production and an increase in eNOS expression [11], [57]. However, it has also been reported that under inflammatory conditions there is an increase in iNOS expression and iNOS associated NO production from endothelial cells [13], [14]. Thus, a prolonged exposure of the endothelium to high concentrations of peroxynitrite and leukocyte activation would eventually increase endothelial O_2_^•−^ production rate. In this scenario, the endothelial O_2_^•−^ production rate may equal or exceed the endothelial NO production progressing the Case 3 to the severe oxidative stress condition (Case 5).

**Figure 11:**
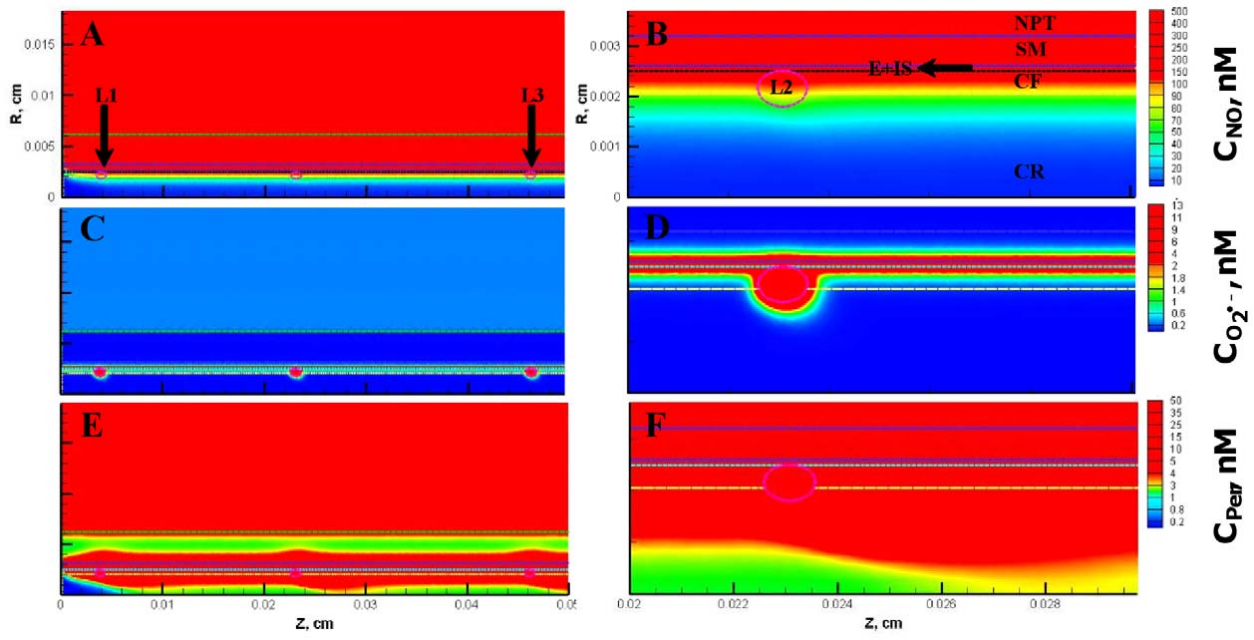
Concentration distribution under severe oxidative stress conditions (Case 5). The NO, O_2_^•−^ and peroxynitrite concentration distribution are shown for the entire arteriolar geometry in **Panels A, C, and E,** respectively and across the 200–300 µm region in **Panels B, D and F,** respectively. The O_2_^•−^ production in the endothelium and capillary in this case were equal to their respective NO production and the leukocytes were in activated state producing NO and O_2_^•−^.

**Figure 12:**
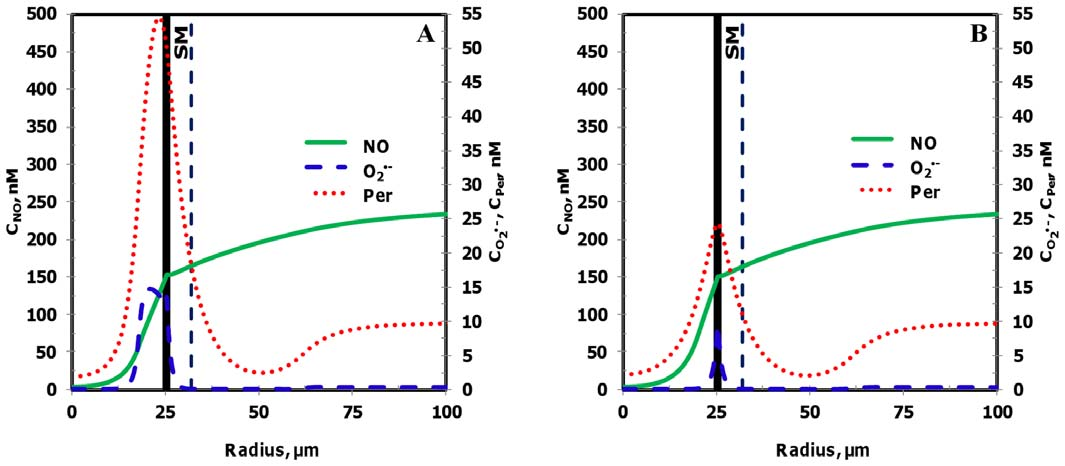
Radial concentration profiles at locations P_1_ and P_2_ for the Case 5. **Panel A** **and** **B** shows the radial concentration profiles of NO, O_2_^•−^ and peroxynitrite at the location P_1_ and P_2_, respectively.

Figures 11 and 12 show the resulting concentration distributions and radial profiles, respectively. In comparison to the Case 3, the NO concentration reduced at all regions of the blood vessel and the leukocytes. Within the leukocytes for the Case 5, the concentration range for NO, O_2_^•−^ and peroxynitrite were 56−144 nM, 6500−14800 pM and 26−56 nM, respectively. The maximum NO concentration changed by 0.5 fold while the maximum O_2_^•−^ and peroxynitrite increased by 1.1 fold in comparison to the Case 3. In comparison to the Case 3, the maximum NO concentration changed by 0.6 fold and peroxynitrite concentrations increased by 1.3 fold across the CR, CF, E and SM regions. The O_2_^•−^ concentration increased by 1.1−3 fold in the CR, CF, E and SM regions, respectively (See Table 5).

Furthermore at the locations P_1_ and P_2_, the maximum endothelial NO concentration changed by 0.6 fold in comparison to the Case 3. The O_2_^•−^ and peroxynitrite concentration reached their respective maximum values of 14672 pM and 55 nM at distances of 21.0 and 23.5 µm, respectively from the center of the vessel at the location P_1_. The maximum O_2_^•−^ concentrations increased by 2 and 6 fold and the maximum peroxynitrite concentrations increased by 1.2 and 4 fold at the locations P_1_ and P_2_, respectively. Thus, the severe oxidative stress condition (Case5) leads to a decrease in NO concentration across all regions of the arteriole and a significant increase in oxidative and nitrosative stress at regions of the arteriole previously unaffected under conditions presented in the Case 2 and 3, respectively (regions located between the centers of the leukocytes).

## Discussion

There is a lack of sufficient measurement data of all these free radical levels in one study to validate the findings from our study. The modeling approach provides us improved understanding on the quantitative aspects of the complex interactions of NO and oxidative stress in the microcirculation. Overall, the results predicted by our models show similar trends to experimental observations as discussed below.

### Limitations of Current Study

The model provides insight into the free radical levels changes during endothelial–leukocyte interactions from normal to oxidative stress state. The model predictions supported previous experimental observations about the independence between leukocyte adhesion and activation as described later. This modeling study has certain limitations. The results may vary depending on 1) the change in the blood vessel diameter due to vasoregulation, 2) the change in blood flow and velocity profile in the presence of endothelium interacting leukocytes, 3) the leukocyte migration and the presence of a nearby leukocyte. These limitations can be addressed by solving the multi-phase momentum transport equation involving blood and leukocytes [58] simultaneously with the mass transport equations of the free radical species shown in equation (1). Additionally, moving boundary conditions need to be introduced at the interfaces separating adjacent regions of the vascular geometry. Such analysis will be extremely complex and will require enormous computational resources.

### Transition from Normal Physiologic Conditions (Case 1) to Oxidative Stress Conditions (Case 2, 3 and 5) Leads to Significant Changes in Free Radical Profiles

Upon transition from normal physiology (Case 1) to endothelial oxidative stress conditions (Case 2), the results showed that the maximum O_2_^•−^ and peroxynitrite concentrations increased by 4 fold in the endothelial and smooth muscle region, respectively. Using DHE fluorescence, Gupte et al. [59] reported a 2 fold increase in O_2_^•−^ levels in the diabetic aortas as compared to aortas of healthy mice. Nagaoka et al. [60] showed that porcine arterioles incubated with a pro-inflammatory marker CRP caused a marked increase in O_2_^•−^ levels in the endothelium compared to untreated arterioles. They did not quantify the level of increase. El-Remessy and co-workers [61] reported a 1.4 fold increase in nitrotyrosine levels, which is an indirect indicator of peroxynitrite levels, for diabetic rat aortas.

The NO concentration changed by 0.9 fold in the endothelial and smooth muscle region in our simulations for the Case 2 in comparison to the Case 1. Akamine et al. [62] estimated the NO levels in mesenteric arterioles of normal and diabetic rats using DAF fluorescence. They reported approximately 0.8 fold change in NO levels of diabetic arterioles compared to normal arterioles. On the contrary, Jebelovszki et al. [63] showed that the endothelial dependent vasodilation in arterioles of lean and obese rats were similar even though oxidative stress increased in vessels of obese rats. The NO concentration range for endothelial oxidative stress condition in the smooth muscle region predicted by this study was 86−266 nM. This is within NO levels required for half maximal activation of sGC (23−100 nM) [35].

In addition, the results from this study predicted that in comparison to normal physiology (Case 1), the combination of endothelial oxidative stress and leukocyte activation (Case 3) changed the maximum NO concentration in the E and SM regions by 0.8 fold whereas the maximum O_2_^•−^ and peroxynitrite concentrations increased by 10−26 fold. The maximum O_2_^•−^ and peroxynitrite concentration in the cell-rich region increased by 1067 and 33 fold, respectively compared to normal physiology. Zhang et al. [64] compared the levels of O_2_^•−^ between normal and TNF-α treated porcine coronary arterioles using DHE fluorescence and observed augmented levels of O_2_^•−^ in the endothelial and smooth muscle region. Suematsu et al. [65] showed that an infusion of rat venules with NOS inhibitor L-NAME and leukocyte activation agent fMLP (formyl-Met-Leu-Phe-OH) caused a 9 fold increase in endothelium ROS concentration. In a study involving rat microvasculature, it was observed that treatment with PAF antagonist reduced the ROS levels in the microvasculature by a factor of 0.6 [66].

Some of the above observations may lack the sufficient measurement data from the system that leads to disagreement in the modeling predictions and experimental observations. Possible explanation for the discrepancy in the extent of changes of free radical levels include higher O_2_^•−^ production and lower NO production in experimental conditions compared to the NO and O_2_^•−^ production rates used in our model. This could be due to activation of several sources of O_2_^•−^ production, inhibition or activation of NO production sources and differences in morphology of the endothelial cells. Additionally, oxidation of DHE by oxidizing agents other than O_2_^•−^ [67], [68], measurement of change in total ROS as opposed to just change in O_2_^•−^ and inhibition of DAF oxidation by antioxidants such as glutathione (GSH) and ascorbate [68] can also contribute to the disagreement between modeling and experimental results.

### Consequences of Increased Oxidative Stress

Simulation of the severe oxidative stress condition (Case 5) using the computational model in this study predicted a significant decrease in the NO concentration and a significant increase in O_2_^•−^ and peroxynitrite concentration in the entire endothelial and smooth muscle region from the levels observed in the Case 3. This was more prominent between the leukocytes in the E and SM regions for severe oxidative stress, whereas the O_2_^•−^ and peroxynitrite concentration for Case 2 and 3 were similar (refer Table 4). A comparison between severe oxidative stress condition (Case 5) and normal physiologic condition (Case 1) indicated that the maximum concentrations of NO changed by 0.6 fold, O_2_^•−^ increased by 27 and 28 fold, and peroxynitrite increased by 31 and 30 fold in the E and SM region, respectively. An increase in O_2_^•−^ and peroxynitrite concentration and a decrease in NO concentration in the vasculature has been implicated for vascular complications resulting from leukocyte-endothelial cell interactions [3], [7], [23], [69].

The reduction in NO concentration in the SM region can severely impair vasodilation. Abu Nabah et al. [70] reported that arterioles of rats injected with eNOS inhibitor L-NAME showed a reduction in shear rate by a factor of 0.5, possibly due to reduced vasodilation. The increase in peroxynitrite concentration in the SM region can cause inhibition of smooth muscle cell growth and leads to their apoptosis. Huang et al. [71] reported that a 5 fold increase in peroxynitrite concentration in smooth muscle cells causes their numbers to reduce by a factor of 0.16. Increase of O_2_^•−^ concentration in the endothelium could lead to deregulation of vascular permeability. Casillan et al. [66] observed a 10 fold increase in vascular permeability when the ROS concentration in the endothelium increased 1.6 fold. Therefore severe oxidative stress conditions may cause impairment of vasodilation, deregulation of vascular permeability, leukocyte transmigration to the SM region and apoptosis of smooth muscle cells. These could eventually lead to complications such as tissue edema and multiple organ failure [5], [53].

### Possible Effect on Leukocytes Because of the Transition from Normal Physiological Condition to the Endothelial Oxidative Stress Condition

The maximum peroxynitrite concentration within the leukocytes and in the CR regions increased approximately by 4 fold upon transition from normal physiological condition (Case 1) to the endothelial oxidative stress condition (Case 2). Rohn et al. [19] reported that peroxynitrite is one of the priming agents for leukocytes. An increase in peroxynitrite concentration within leukocytes can prime them by upregulation of secondary stimulus receptors on their surface, depolarization of their mitochondrial membrane, increase in actin polymerization, enhancement of NADPH oxidase activity and increase in their intracellular [Ca^2+^] concentration [19], [72], [73]. Additionally, the maximum O_2_^•−^ concentration within the leukocytes and the CR regions increased by 5 and 4 fold, respectively upon transition from the Case 1 to 2. The increased concentration of O_2_^•−^ in the lumen through its dismutation product H_2_O_2_ can serve as secondary stimuli to the already primed circulating leukocytes within the lumen and activate them to produce NO and O_2_^•−^ without any direct interaction with the endothelium [6], [7]. Thus, priming and activation can also occur for leukocytes circulating in the lumen in oxidative stress conditions. This is in agreement with earlier studies that have shown that leukocyte adhesion to the endothelium and activation of leukocytes are two completely independent events [25], [74]. Additionally, the increase in peroxynitrite concentration within the leukocytes and the vasculature can cause tyrosine nitration, nitrosylation of various proteins, DNA strand breakage, increase in gene expression such as TNF-α, IL-6, IL-1β, ICAM-1 and P-selectin, and changes in signaling pathways such as MAP kinase, protein kinase C and NF-kβ, inactivation of anti-oxidant SOD, cell necrosis and cell apoptosis [4], [69], [75].

Comparison of the simulation results in this study between normal physiological condition (Case 1) with that of the combination of endothelial oxidative stress and leukocyte activation (Case 3) shows that the maximum NO concentration in the leukocytes reduced by a factor of 0.9 while the maximum O_2_^•−^ and peroxynitrite concentration increased by 70 and 31 fold, respectively. Chemiluminescence analysis by Zhu et al. [25] on fMLP stimulated neutrophils indicated a 6 fold increase in their ROS concentration. Gagnon et al. [76] used DHR 123 (dihydrorhodamine 123) based fluorescence analysis and reported a 2.4 fold increase in peroxynitrite concentration in LPS (lipopolysaccharide) treated granulocytes as compared to normal granulocytes. Information regarding measurement of changes in leukocyte NO concentration during activation of leukocytes is insufficient. However, it is reported that inhibition of leukocyte based NO production increases the expression of adhesion molecules on the leukocyte surface and promotes adhesion of leukocytes to the vessel wall [77].

If the circulating leukocytes are monocytes then the increased O_2_^•−^ and peroxynitrite concentration within the monocytes may cause them to transform to macrophages [78], [79]. Macrophages can transmigrate into the SM region causing proliferation of smooth muscle cells, ROS production from SM region, tissue injury and eventual vascular remodeling [23], [53]. The excess peroxynitrite within the leukocytes can contribute to tissue injury by protein tyrosine nitration [80]. However, certain studies have argued against the role of peroxynitrite in tyrosine nitration of proteins and have attributed it to nitrite formed from oxidation of NO [81], [82]. Our study suggests the role of peroxynitrite in tyrosine nitration as it takes into account both the oxidation reaction of NO and the reaction between NO and O_2_^•−^ to form peroxynitrite.

### Importance of SOD in Reduction of O_2_^•−^ and Peroxynitrite Concentrations During Endothelial Oxidative Stress Coupled with Leukocyte Activation

Several studies have shown SOD to be an effective therapeutic agent for treatment of leukocyte-endothelial cell interaction based vascular complications [83], [84], [85]. Additionally an increase in SOD expression and activity was reported during endothelial dysfunction [85], [86]. We wanted to test the effectiveness of SOD as a therapeutic through our model. We observed a significant decrease in the O_2_^•−^ and peroxynitrite levels and an increase in the NO levels in the different regions of the vasculature and the leukocytes upon increasing the concentration of SOD from 1 to 10 µM across all the regions of the vessel and within the leukocytes under conditions of combined endothelial oxidative stress and leukocyte activation (transition from the Case 3 to the Case 4). Zhu et al. [25] compared the ROS production using chemiluminescence between untreated and SOD incubated neutrophils with both subjected to fMLP stimulation and observed that ROS levels in the SOD incubated neutrophils reduced by a factor of 0.3.

Zhu et al. [25] also reported that perfusion of venules with fMLP stimulated neutrophils caused an increase in intracellular Ca^2+^ concentration in endothelial cells, which was reduced by a factor of 0.7 upon perfusion with SOD. Bertuglia and Giusti [27] showed that microvessels subjected to ischemia reperfusion had 2 fold increase in ROS production and a decrease in RBC flow velocity by a factor of 0.9. However, ROS production and RBC flow velocity did not increase when these microvessels were treated with SOD. Murohara et al. [33] reported a significant reduction in vasoconstriction and leukocyte adhesion to the endothelium in arteries subjected to oxidative stress upon treatment of superoxide dismutase (SOD) and nitric oxide synthase (NOS) inhibitors. However, H_2_O_2_ and hydroxyl radial scavengers catalase and N-2-mercaptopropionyl-glycine (MPG), respectively had minimal effect on vasoconstriction and leukocyte adhesion.

Another qualitative study involving DHE-fluorescence imaging of TNF-α incubated arterioles showed a significant increase in O_2_^•−^ concentration in the E and SM region, which was considerably reduced upon addition of SOD mimetic Tempol [64]. In the lumen, the maximum O_2_^•−^ and peroxynitrite concentrations reduced by a factor of 0.1 and 0.2, respectively while the NO concentration increased by 1.2 fold. This could prevent the possible priming and activation of leukocytes circulating in the lumen and also prevent their recruitment towards the endothelium [6], [7], [77]. Thus, the scavenging of O_2_^•−^ by SOD plays a key role in reducing peroxynitrite concentration, increasing NO bioavailability in SM region for vasodilation and prevention of leukocyte rolling, adhesion and activation [8], [25], [87], [88]. SOD seems to be a promising candidate as potential therapeutic for treatment of vascular complications arising as a result of leukocyte-endothelial cell interactions.

**In conclusion,** the model developed in this study predicts the effects of the presence of leukocytes along the endothelium on NO, O_2_^•−^ and peroxynitrite concentration distribution in the microcirculation under several endothelial oxidative stress states. The results indicate that the onset of endothelial oxidative stress causes an increase in both O_2_^•−^ and peroxynitrite concentration within the leukocytes and the lumen, which can help prime (through peroxynitrite) and activate (secondary stimuli through O_2_^•−^) the leukocytes along the endothelium and those circulating in the lumen. The results thus provide an explanation to the previous experimental findings that leukocyte rolling/adhesion and activation are completely independent events and also explains the limited success of anti-adhesion therapies used to prevent vascular dysfunction [7]. The results show that the oxidative and nitrosative stress during the leukocyte-endothelial cell interactions may possibly contribute to microvascular dysfunction and tissue injury.

## References

[1] Chow FY, Nikolic-Paterson DJ, Ozols E, Atkins RC, Tesch GH (2005). Intercellular adhesion molecule-1 deficiency is protective against nephropathy in type 2 diabetic db/db mice.. J Am Soc Nephrol.

[2] Geraldes P, King GL (2010). Activation of protein kinase C isoforms and its impact on diabetic complications.. Circ Res.

[3] Al-Shabrawey M, Rojas M, Sanders T, Behzadian A, El-Remessy A (2008). Role of NADPH oxidase in retinal vascular inflammation.. Invest Ophthalmol Vis Sci.

[4] Julius U, Drel VR, Grassler J, Obrosova IG (2009). Nitrosylated proteins in monocytes as a new marker of oxidative-nitrosative stress in diabetic subjects with macroangiopathy.. Exp Clin Endocrinol Diabetes.

[5] Sumagin R, Lomakina E, Sarelius IH (2008). Leukocyte-endothelial cell interactions are linked to vascular permeability via ICAM-1-mediated signaling.. Am J Physiol Heart Circ Physiol.

[6] He P, Zhang H, Zhu L, Jiang Y, Zhou X (2006). Leukocyte-platelet aggregate adhesion and vascular permeability in intact microvessels: role of activated endothelial cells.. Am J Physiol Heart Circ Physiol.

[7] He P (2010). Leucocyte/endothelium interactions and microvessel permeability: coupled or uncoupled?. Cardiovasc Res.

[8] Kubes P, Suzuki M, Granger DN (1990). Modulation of PAF-induced leukocyte adherence and increased microvascular permeability.. Am J Physiol.

[9] Wang Q, Doerschuk CM (2000). Neutrophil-induced changes in the biomechanical properties of endothelial cells: roles of ICAM-1 and reactive oxygen species.. J Immunol.

[10] Vedder NB, Winn RK, Rice CL, Chi EY, Arfors KE (1990). Inhibition of leukocyte adherence by anti-CD18 monoclonal antibody attenuates reperfusion injury in the rabbit ear.. Proc Natl Acad Sci U S A.

[11] Hink U, Li H, Mollnau H, Oelze M, Matheis E (2001). Mechanisms underlying endothelial dysfunction in diabetes mellitus.. Circ Res.

[12] Paolocci N, Biondi R, Bettini M, Lee CI, Berlowitz CO (2001). Oxygen radical-mediated reduction in basal and agonist-evoked NO release in isolated rat heart.. J Mol Cell Cardiol.

[13] Wilcox JN, Subramanian RR, Sundell CL, Tracey WR, Pollock JS (1997). Expression of multiple isoforms of nitric oxide synthase in normal and atherosclerotic vessels.. Arterioscler Thromb Vasc Biol.

[14] Steiner L, Kroncke K, Fehsel K, Kolb-Bachofen V (1997). Endothelial cells as cytotoxic effector cells: cytokine-activated rat islet endothelial cells lyse syngeneic islet cells via nitric oxide.. Diabetologia.

[15] Sumagin R, Sarelius IH (2007). A role for ICAM-1 in maintenance of leukocyte-endothelial cell rolling interactions in inflamed arterioles.. Am J Physiol Heart Circ Physiol.

[16] Thorlacius H, Lindbom L, Raud J (1997). Cytokine-induced leukocyte rolling in mouse cremaster muscle arterioles in P-selectin dependent.. Am J Physiol.

[17] Sumagin R, Sarelius IH (2006). TNF-alpha activation of arterioles and venules alters distribution and levels of ICAM-1 and affects leukocyte-endothelial cell interactions.. Am J Physiol Heart Circ Physiol.

[18] Hayashi T, Juliet PA, Miyazaki A, Ignarro LJ, Iguchi A (2007). High glucose downregulates the number of caveolae in monocytes through oxidative stress from NADPH oxidase: implications for atherosclerosis.. Biochim Biophys Acta.

[19] Rohn TT, Nelson LK, Sipes KM, Swain SD, Jutila KL (1999). Priming of human neutrophils by peroxynitrite: potential role in enhancement of the local inflammatory response.. J Leukoc Biol.

[20] Bellavite P (1988). The superoxide-forming enzymatic system of phagocytes.. Free Radic Biol Med.

[21] Mastej K, Adamiec R (2008). Neutrophil surface expression of CD11b and CD62L in diabetic microangiopathy.. Acta Diabetol.

[22] Zheng L, Du Y, Miller C, Gubitosi-Klug RA, Ball S (2007). Critical role of inducible nitric oxide synthase in degeneration of retinal capillaries in mice with streptozotocin-induced diabetes.. Diabetologia.

[23] Csanyi G, Taylor WR, Pagano PJ (2009). NOX and inflammation in the vascular adventitia.. Free Radic Biol Med.

[24] Granger DN (1988). Role of xanthine oxidase and granulocytes in ischemia-reperfusion injury.. Am J Physiol.

[25] Zhu L, Castranova V, He P (2005). fMLP-stimulated neutrophils increase endothelial [Ca2+]i and microvessel permeability in the absence of adhesion: role of reactive oxygen species.. Am J Physiol Heart Circ Physiol.

[26] Sarelius IH, Kuebel JM, Wang J, Huxley VH (2006). Macromolecule permeability of in situ and excised rodent skeletal muscle arterioles and venules.. Am J Physiol Heart Circ Physiol.

[27] Bertuglia S, Giusti A (2003). Microvascular oxygenation, oxidative stress, NO suppression and superoxide dismutase during postischemic reperfusion.. Am J Physiol Heart Circ Physiol.

[28] Jariyapongskul A, Rungjaroen T, Kasetsuwan N, Pathumraj S, Niimi H (2006). Chronic changes of the iris microvasculature of streptozotocin-induced diabetic rats using fluorescence videomicroscopy.. Clin Hemorheol Microcirc.

[29] Kunkel EJ, Jung U, Ley K (1997). TNF-alpha induces selectin-mediated leukocyte rolling in mouse cremaster muscle arterioles.. Am J Physiol.

[30] Gujral JS, Hinson JA, Farhood A, Jaeschke H (2004). NADPH oxidase-derived oxidant stress is critical for neutrophil cytotoxicity during endotoxemia.. Am J Physiol Gastrointest Liver Physiol.

[31] Popel AS (1989). Theory of oxygen transport to tissue.. Crit Rev Biomed Eng.

[32] Okamoto E, Couse T, De Leon H, Vinten-Johansen J, Goodman RB (2001). Perivascular inflammation after balloon angioplasty of porcine coronary arteries.. Circulation.

[33] Murohara T, Buerke M, Lefer AM (1994). Polymorphonuclear leukocyte-induced vasocontraction and endothelial dysfunction. Role of selectins.. Arterioscler Thromb.

[34] Kavdia M, Popel AS (2006). Venular endothelium-derived NO can affect paired arteriole: a computational model.. Am J Physiol Heart Circ Physiol.

[35] Kavdia M, Popel AS (2004). Contribution of nNOS- and eNOS-derived NO to microvascular smooth muscle NO exposure.. J Appl Physiol.

[36] Kavdia M, Popel AS (2003). Wall shear stress differentially affects NO level in arterioles for volume expanders and Hb-based O2 carriers.. Microvasc Res.

[37] Coulter NA, Pappenheimer JR (1949). Development of turbulence in flowing blood.. Am J Physiol.

[38] Chen X, Jaron D, Barbee KA, Buerk DG (2006). The influence of radial RBC distribution, blood velocity profiles, and glycocalyx on coupled NO/O2 transport.. J Appl Physiol.

[39] Kavdia M, Tsoukias NM, Popel AS (2002). Model of nitric oxide diffusion in an arteriole: impact of hemoglobin-based blood substitutes.. Am J Physiol Heart Circ Physiol.

[40] Deonikar P, Kavdia M (2010). A computational model for nitric oxide, nitrite and nitrate biotransport in the microcirculation: effect of reduced nitric oxide consumption by red blood cells and blood velocity.. Microvasc Res.

[41] Kavdia M (2006). A computational model for free radicals transport in the microcirculation.. Antioxid Redox Signal.

[42] Forstermann U, Munzel T (2006). Endothelial nitric oxide synthase in vascular disease: from marvel to menace.. Circulation.

[43] Malinski T, Taha Z, Grunfeld S, Patton S, Kapturczak M (1993). Diffusion of nitric oxide in the aorta wall monitored in situ by porphyrinic microsensors.. Biochem Biophys Res Commun.

[44] Vaughn MW, Kuo L, Liao JC (1998). Effective diffusion distance of nitric oxide in the microcirculation.. Am J Physiol.

[45] Nalwaya N, Deen WM (2004). Analysis of the effects of nitric oxide and oxygen on nitric oxide production by macrophages.. J Theor Biol.

[46] Tsukimori K, Fukushima K, Tsushima A, Nakano H (2005). Generation of reactive oxygen species by neutrophils and endothelial cell injury in normal and preeclamptic pregnancies.. Hypertension.

[47] Ting-Beall HP, Needham D, Hochmuth RM (1993). Volume and osmotic properties of human neutrophils.. Blood.

[48] Ellsworth ML, Popel AS, Pittman RN (1988). Assessment and impact of heterogeneities of convective oxygen transport parameters in capillaries of striated muscle: experimental and theoretical.. Microvasc Res.

[49] Damiano ER, Westheider J, Tozeren A, Ley K (1996). Variation in the velocity, deformation, and adhesion energy density of leukocytes rolling within venules.. Circ Res.

[50] Nalwaya N, Deen WM (2003). Analysis of cellular exposure to peroxynitrite in suspension cultures.. Chem Res Toxicol.

[51] Kubes P, Kerfoot SM (2001). Leukocyte recruitment in the microcirculation: the rolling paradigm revisited.. News Physiol Sci.

[52] Zou MH (2007). Peroxynitrite and protein tyrosine nitration of prostacyclin synthase.. Prostaglandins Other Lipid Mediat.

[53] Galkina E, Ley K (2006). Leukocyte recruitment and vascular injury in diabetic nephropathy.. J Am Soc Nephrol.

[54] Hubbard AK, Rothlein R (2000). Intercellular adhesion molecule-1 (ICAM-1) expression and cell signaling cascades.. Free Radic Biol Med.

[55] Deonikar P, Kavdia M (2010). An integrated computational and experimental model of nitric oxide-red blood cell interactions.. Ann Biomed Eng.

[56] Buerk DG, Lamkin-Kennard K, Jaron D (2003). Modeling the influence of superoxide dismutase on superoxide and nitric oxide interactions, including reversible inhibition of oxygen consumption.. Free Radic Biol Med.

[57] Kar S, Kavdia M (2011). Modeling of biopterin-dependent pathways of eNOS for nitric oxide and superoxide production.. Free Radic Biol Med.

[58] Lyczkowski RW, Alevriadou BR, Horner M, Panchal CB, Shroff SG (2009). Application of multiphase computational fluid dynamics to analyze monocyte adhesion.. Ann Biomed Eng.

[59] Gupte S, Labinskyy N, Gupte R, Csiszar A, Ungvari Z (2010). Role of NAD(P)H oxidase in superoxide generation and endothelial dysfunction in Goto-Kakizaki (GK) rats as a model of nonobese NIDDM.. PLoS One.

[60] Nagaoka T, Kuo L, Ren Y, Yoshida A, Hein TW (2008). C-reactive protein inhibits endothelium-dependent nitric oxide-mediated dilation of retinal arterioles via enhanced superoxide production.. Invest Ophthalmol Vis Sci.

[61] El-Remessy AB, Tawfik HE, Matragoon S, Pillai B, Caldwell RB (2010). Peroxynitrite mediates diabetes-induced endothelial dysfunction: possible role of Rho kinase activation.. Exp Diabetes Res.

[62] Akamine EH, Kawamoto EM, Scavone C, Nigro D, Carvalho MH (2006). Correction of endothelial dysfunction in diabetic female rats by tetrahydrobiopterin and chronic insulin.. J Vasc Res.

[63] Jebelovszki E, Kiraly C, Erdei N, Feher A, Pasztor ET (2008). High-fat diet-induced obesity leads to increased NO sensitivity of rat coronary arterioles: role of soluble guanylate cyclase activation.. Am J Physiol Heart Circ Physiol.

[64] Zhang C, Hein TW, Wang W, Ren Y, Shipley RD (2006). Activation of JNK and xanthine oxidase by TNF-alpha impairs nitric oxide-mediated dilation of coronary arterioles.. J Mol Cell Cardiol.

[65] Suematsu M, Tamatani T, Delano FA, Miyasaka M, Forrest M (1994). Microvascular oxidative stress preceding leukocyte activation elicited by in vivo nitric oxide suppression.. Am J Physiol.

[66] Casillan AJ, Gonzalez NC, Johnson JS, Steiner DR, Wood JG (2003). Mesenteric microvascular inflammatory responses to systemic hypoxia are mediated by PAF and LTB4.. J Appl Physiol.

[67] Zhao H, Kalivendi S, Zhang H, Joseph J, Nithipatikom K (2003). Superoxide reacts with hydroethidine but forms a fluorescent product that is distinctly different from ethidium: potential implications in intracellular fluorescence detection of superoxide.. Free Radic Biol Med.

[68] Wardman P (2007). Fluorescent and luminescent probes for measurement of oxidative and nitrosative species in cells and tissues: progress, pitfalls, and prospects.. Free Radic Biol Med.

[69] Szabo C (2003). Multiple pathways of peroxynitrite cytotoxicity.. Toxicol Lett.

[70] Nabah YN, Mateo T, Cerda-Nicolas M, Alvarez A, Martinez M (2005). L-NAME induces direct arteriolar leukocyte adhesion, which is mainly mediated by angiotensin-II.. Microcirculation.

[71] Huang J, Lin SC, Nadershahi A, Watts SW, Sarkar R (2008). Role of redox signaling and poly (adenosine diphosphate-ribose) polymerase activation in vascular smooth muscle cell growth inhibition by nitric oxide and peroxynitrite.. J Vasc Surg.

[72] Zimmerli W, Seligmann B, Gallin JI (1986). Exudation primes human and guinea pig neutrophils for subsequent responsiveness to the chemotactic peptide N-formylmethionylleucylphenylalanine and increases complement component C3bi receptor expression.. J Clin Invest.

[73] Adrie C, Richter C, Bachelet M, Banzet N, Francois D (2000). Contrasting effects of NO and peroxynitrites on HSP70 expression and apoptosis in human monocytes.. Am J Physiol Cell Physiol.

[74] Zeng M, Zhang H, Lowell C, He P (2002). Tumor necrosis factor-alpha-induced leukocyte adhesion and microvessel permeability.. Am J Physiol Heart Circ Physiol.

[75] Pacher P, Beckman JS, Liaudet L (2007). Nitric oxide and peroxynitrite in health and disease.. Physiol Rev.

[76] Gagnon C, Leblond FA, Filep JG (1998). Peroxynitrite production by human neutrophils, monocytes and lymphocytes challenged with lipopolysaccharide.. FEBS Lett.

[77] Liu P, Yin K, Nagele R, Wong PY (1998). Inhibition of nitric oxide synthase attenuates peroxynitrite generation, but augments neutrophil accumulation in hepatic ischemia-reperfusion in rats.. J Pharmacol Exp Ther.

[78] Lu J, Mitra S, Wang X, Khaidakov M, Mehta JL (2011). Oxidative stress and lectin-like ox-LDL-receptor LOX-1 in atherogenesis and tumorigenesis.. Antioxid Redox Signal.

[79] Fuhrman B, Shiner M, Volkova N, Aviram M (2004). Cell-induced copper ion-mediated low density lipoprotein oxidation increases during in vivo monocyte-to-macrophage differentiation.. Free Radic Biol Med.

[80] Beckman JS, Koppenol WH (1996). Nitric oxide, superoxide, and peroxynitrite: the good, the bad, and ugly.. Am J Physiol.

[81] Pfeiffer S, Lass A, Schmidt K, Mayer B (2001). Protein tyrosine nitration in cytokine-activated murine macrophages. Involvement of a peroxidase/nitrite pathway rather than peroxynitrite.. J Biol Chem.

[82] Pfeiffer S, Mayer B (2002). Protein tyrosine nitration and peroxynitrite: reply.. FASEB J.

[83] Segui J, Gil F, Gironella M, Alvarez M, Gimeno M (2005). Down-regulation of endothelial adhesion molecules and leukocyte adhesion by treatment with superoxide dismutase is beneficial in chronic immune experimental colitis.. Inflamm Bowel Dis.

[84] Feng L, Ke N, Cheng F, Guo Y, Li S (2011). The protective mechanism of ligustrazine against renal ischemia/reperfusion injury.. J Surg Res.

[85] Yang H, Zhou L, Wang Z, Roberts LJ, Lin X (2009). Overexpression of antioxidant enzymes in ApoE-deficient mice suppresses benzo(a)pyrene-accelerated atherosclerosis.. Atherosclerosis.

[86] Ceriello A, dello Russo P, Amstad P, Cerutti P (1996). High glucose induces antioxidant enzymes in human endothelial cells in culture. Evidence linking hyperglycemia and oxidative stress.. Diabetes.

[87] Frisbee JC, Stepp DW (2001). Impaired NO-dependent dilation of skeletal muscle arterioles in hypertensive diabetic obese Zucker rats.. Am J Physiol Heart Circ Physiol.

[88] Kaul DK, Liu XD, Choong S, Belcher JD, Vercellotti GM (2004). Anti-inflammatory therapy ameliorates leukocyte adhesion and microvascular flow abnormalities in transgenic sickle mice.. Am J Physiol Heart Circ Physiol.

[89] Potdar S, Kavdia M (2009). NO/peroxynitrite dynamics of high glucose-exposed HUVECs: chemiluminescent measurement and computational model.. Microvasc Res.

